# Regulation of Na^+^/K^+^ ATPase Transport Velocity by RNA Editing

**DOI:** 10.1371/journal.pbio.1000540

**Published:** 2010-11-23

**Authors:** Claudia Colina, Juan Pablo Palavicini, Deepa Srikumar, Miguel Holmgren, Joshua J. C. Rosenthal

**Affiliations:** 1 Institute of Neurobiology, University of Puerto Rico–Medical Sciences Campus, San Juan, Puerto Rico; 2 Molecular Neurophysiology Section, Porter Neuroscience Research Center, National Institute of Neurological Disorders and Stroke, National Institutes of Health, Bethesda, Maryland, United States of America; 3 Department of Biochemistry, University of Puerto Rico–Medical Sciences Campus, San Juan, Puerto Rico; Brown, United States of America

## Abstract

Because firing properties and metabolic rates vary widely, neurons require different transport rates from their Na^+^/K^+^ pumps in order to maintain ion homeostasis. In this study we show that Na^+^/K^+^ pump activity is tightly regulated by a novel process, RNA editing. Three codons within the squid Na^+^/K^+^ ATPase gene can be recoded at the RNA level, and the efficiency of conversion for each varies dramatically, and independently, between tissues. At one site, a highly conserved isoleucine in the seventh transmembrane span can be converted to a valine, a change that shifts the pump's intrinsic voltage dependence. Mechanistically, the removal of a single methyl group specifically targets the process of Na^+^ release to the extracellular solution, causing a higher turnover rate at the resting membrane potential.

## Abstract 2

Editing of Na^+^/K^+^ ATPase mRNAs modulates the Na^+^/K^+^ pump's turnover rate by selectively targeting the release of the final sodium to the outside.

## Author Summary

In order for excitable cells like neurons and muscles to generate electrical signals, they require ion gradients across their plasma membranes. For example, sodium concentrations are much lower inside a cell than outside, and for potassium it is the opposite case. The job of maintaining these ion gradients falls squarely on a single protein: the Na^+^/K^+^ pump. During each transport cycle, this enzyme moves three sodium ions out of the cell and imports two of potassium. Because this process is the foundation for so many physiological processes, the Na^+^/K^+^ pump is costly to operate, using ∼30% of the ATP generated by an organism. Proper regulation of its turnover rate is vital. In this work, we use the giant nerve cell of squid as a model to show that the Na^+^/K^+^ pump can be regulated by an unsuspected mechanism. Although the gene that codes for this enzyme can make a perfectly functional pump, sometimes its information changes as it passes through the messenger RNA. This is achieved by editing RNA and as a result subtly different versions of the pump can be made, differing at only three amino acids out of more than a thousand. We demonstrate that RNA editing modulates the Na^+^/K^+^ pump's turnover rate and sodium release.

## Introduction

Within the animal kingdom, the Na^+^/K^+^ ATPase is a nearly ubiquitous membrane protein that uses the free energy of ATP hydrolysis to establish and maintain the Na^+^ and K^+^ gradients across cell membranes. Na^+^/K^+^ pump activity is essential. Without it cells would lack the driving force required for excitability and Na^+^-coupled transport of solutes in and out of the cell. Because the Na^+^/K^+^ pump is costly to operate, using ∼30% of the ATP generated by an organism [1]–[4], proper regulation of its turnover rate is critical. The Na^+^/K^+^ pump is an electrogenic machine, its activity being directly influenced by the transmembrane potential. Turnover rates are maximal at potentials greater than ∼0 mV and decline steadily at negative potentials. This inhibition results from a combination of effects. High extracellular Na^+^ concentration and negative potentials both tend to drive Na^+^ back to its binding sites deep within the protein's core [5]–[9]. Interestingly, nature has tuned pump activity so that it is inhibited to a similar extent, irrespective of an organism's ionic environment. For example, at the resting potential Na^+^/K^+^ pumps from squid, frogs, or guinea pigs operate at ∼50% activity in the face of drastically different extracellular Na^+^ concentrations [10]–[13]. It is reasonable to hypothesize that by limiting pumping at negative potentials, activity could be upregulated during periods of heightened activity in order to meet the demands of ion homeostasis.

In the nervous system of higher metazoans, RNA editing by adenosine deamination has evolved as an important mechanism for the diversification of the proteome. By removing a single amine that participates in Watson-Crick base-pairing, specific adenosines are converted to inosines within mRNAs and other RNAs. For editing sites within the coding sequence of mRNAs, inosine is read as guanine during translation, causing codons, and protein structure, to change. In both vertebrates and invertebrates, editing targets mRNAs that encode proteins directly involved in action potential conduction and synaptic transmission, and therefore it is assumed that the process is important for regulating rapid electrical signaling [14]–[22]. For some editing sites the specific changes to protein function have been described, however very little is known about their mechanisms of action. In fact, there are just a couple of cases in which we know how edits alter protein function [18],[21]. In this study we show that RNA editing may regulate ion homeostasis by making specific changes within Na^+^/K^+^ pump mRNAs. These changes affect the Na^+^/K^+^ pump's intrinsic voltage dependence. Mechanistically, this is achieved by shifting the occupancy of the states of the transport cycle associated with the release of Na^+^.

## Results

**Figure 1:**
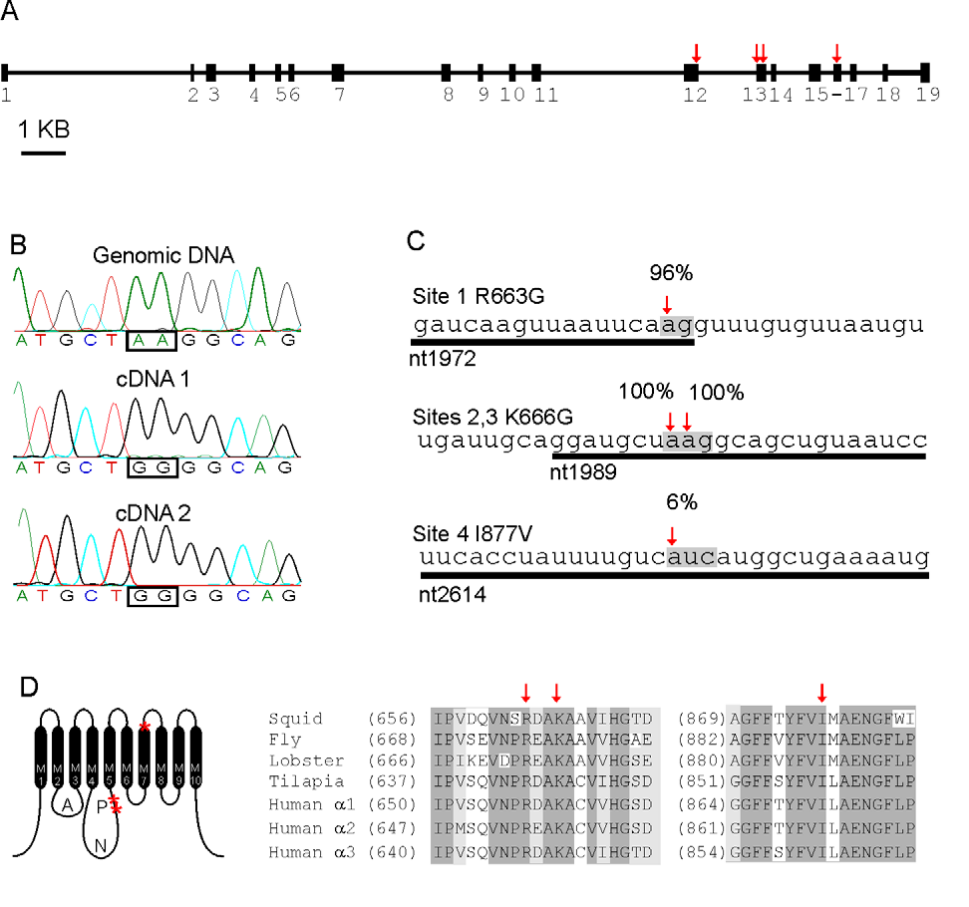
mRNAs for the squid Na^+^/K^+^ pump are edited. (A) A diagram of the intron-exon structure for the SqNaKα1 gene (the cDNA sequence is genbank EF467998). Boxes represent exons and lines introns. Arrows mark the four positions where the gene sequence contained an A and some cDNA sequences contained a G. (B) Electropherogram of the genomic sequence surrounding codon K666 compared to electropherograms of two cDNA clones, which show the first two nucleotides of the codon edited to G, resulting in the codon change K666G. (C) Positions of all four edited adenosines. Numbers refer to the editing percentage based on 50 individual cDNA clones amplified from giant axon system specific cDNA. Exon sequence is underlined. (D) Relative positions of the three codons changed by editing mapped on a cartoon of the Na^+^/K^+^ pump structure. (E) Amino acid alignment of Na^+^/K^+^ pump α subunit sequences from diverse taxa showing that the unedited amino acid at each editing site is highly conserved.

Historically, the Na^+^/K^+^ ATPase of the squid giant axon has been one of the most actively studied native pumps. In a previous report we identified the mRNA sequences for the underlying α (EF467998) and β (EF467996) subunits [10]. Because other squid transcripts are regulated by RNA editing [17],[20], we examined whether the Na^+^/K^+^ ATPase mRNAs were as well. Sequences of 50 individual cDNA clones for the squid NaKα1 subunit, isolated from the giant axon system, showed adenosine-or-guanine variation at specific sites, a hallmark of RNA editing. To explore whether this variation was indeed due to RNA editing, we cloned the gene that encodes squid NaKα1 mRNAs (Figure 1A). The squid NaKα1 gene, which spans over 20 KB, is highly fragmented, containing 19 exons. At four positions, the gene sequence contains an A whereas some or all of the cDNA sequences contain a G (e.g., Figure 1B). Three of the sites lie at the junction with a nearby intron, as is commonly the case with other RNA editing sites (Figure 1C) [23]. Two sites lie within the same codon. Because both were guanosine in all cDNAs sequenced, the lysine at this position was always converted to glycine. To further support the idea that the A→G conversions are caused by RNA editing, we tested whether a squid editing enzyme (SqADAR2.1A (FJ478450.1); [24]) could edit these codons in vitro (Figure S1A). Using the genomic form of the full-length, mature squid NaKα1 mRNA as a substrate, recombinant SqADAR2.1A could edit all four codons. It is notable that all the information required for editing resides within the exons and that intron sequence was not required, as is commonly the case for other editing sites. Similarly, the structure that drives editing of human Kv1.1 channel mRNAs is entirely exonic [18],[25]. Interestingly, human ADAR2 (BC065545.1) can also edit codons K666G and I877V, but not R663G. Predicted folds for NaKα1 mRNA using MFOLD software show an obvious hairpin surrounding the I877V codon (Figure S1B). Using this approach, similar structures are not apparent around codons R663G and K666G. In any case, the combination of our cloning data and the in vitro editing assays verify that the A/G variation observed in Na^+^/K^+^ ATPase mRNAs is due to RNA editing.

The editing sites R663G and K666G are located within the phosphorylation domain which accepts ATP's γ phosphate during the transport cycle, while the I877V edit lies at the extracellular end of the seventh transmembrane segment. All editing sites recode a highly conserved amino acid (R663G, K666G, and I877V; Figure 1D). In fact, a survey of over 200 NaKα1 cDNA sequences from both vertebrates and invertebrates shows the unedited codon at these positions to be almost invariant. There are a few exceptions, however. The ovine and bovine NaKα1 sequences (emb CAA26582.1 and gb AAI23865.1), and a NaK sequence from planaria (dbj BAA32798.1), have an arginine at codon 666, conceivably being produced by editing at the codon's second position (AAR→AGR). At position 877, an electric eel NaK cDNA is the only sequence with a valine. As with squid, this could have been caused by RNA editing. Overall, however, our bioinformatics search uncovered little evidence of editing in distantly related organisms. Because of this we tested whether the squid sites are edited in another cephalopod. Using squid specific primers, NaKα1 cDNA and genomic DNA was amplified from *Octopus bimaculata* collected from Catalina Island, CA. Based on 50 individual cDNA clones, the R663G edit was edited, but at a much lower rate than in Loligo (12% versus 96%). No editing was apparent in codons K666 or I877. These data suggest that editing sites are evolving rapidly within cephalopods.

**Figure 2:**
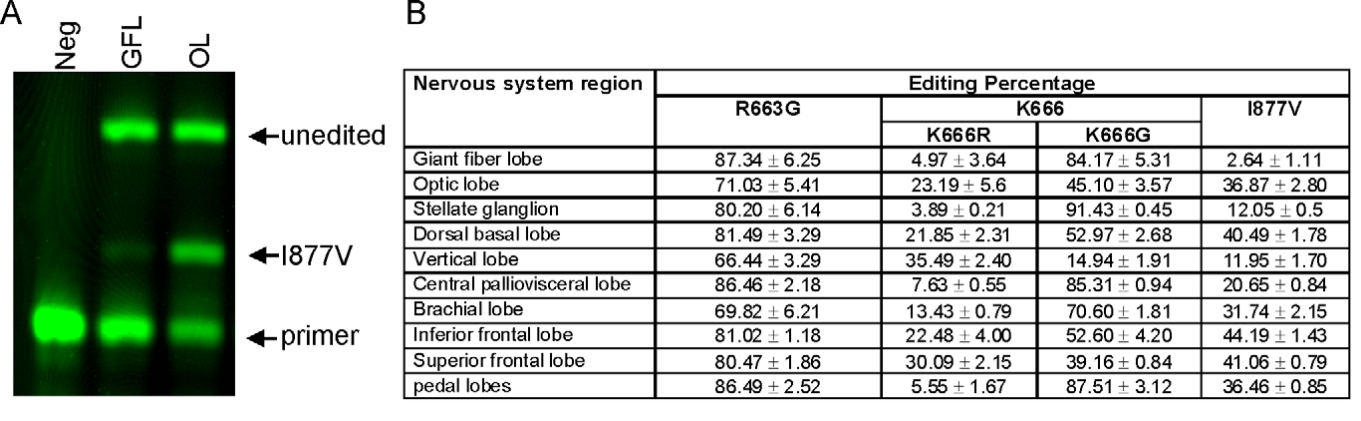
RNA editing efficiency is highly regulated between different areas of the nervous system. (A) An example of the fluorescent poison-primer extension assay [26] used to measure the frequency of the I877V edit in the giant fiber lobe (GFL; giant axon system) and the optic lobe (OL) neurons. (B) Cumulative results from a broad range of tissues (all numbers are expressed as mean ± s.d. *N* = 4 for each tissue). To determine the editing efficiency of all four editing sites in different tissues, the full-length SqNaKα1 cDNA was amplified using cDNA specific to different regions of the *Loligo* nervous system [42],[43] and then used as a template for the assay.

In the giant axon, R663G and K666G are edited almost to completion while I887V is scarcely edited. Why undergo a complex process such as editing when a simple mutation to the gene would produce much the same result? One possibility is that these sites are used for regulating pump function. If this is the case, we would expect the extent of editing at these sites to differ between neuronal tissues. To test this idea, we collected tissue from 10 different regions of the nervous system, both central and peripheral. Using a poison-primer extension assay [26], we estimated the editing efficiency in each sample (Figure 2A). The extent of variation differed dramatically between sites (Figure 2B). R663G varied only from ∼65%–85%. Editing at codon 666 was more complicated. Because it can be incompletely edited at the first two positions (AAG), a mixed population of pumps with either arginine, glycine, or lysine (unedited) can result. In some tissues, as in the giant axon, K666G predominates, while in others K666R or K666 is the dominant species. Although K666E is theoretically possible (GAG), this edit was never observed. The I877V edit is also highly tissue- specific. Barely present in the giant axon system and other peripheral regions, it occurs close to 50% of the time in parts of the central nervous system such as the Inferior Frontal Lobe neurons. These results strongly suggest that RNA editing could be used to regulate Na^+^/K^+^ pump function.

For any cell maintaining ion homeostasis, the most important aspect of Na^+^/K^+^ pump function is the velocity of ion transport. Accordingly, we were interested in determining whether any of the RNA editing events regulates the Na^+^/K^+^ pump's turnover rate. Because the Na^+^/K^+^ pump is electrogenic and its stoichiometry does not change with voltage [12], the pump current (I_p_) is an accurate reflection of the turnover rate at any voltage. Under physiological conditions, the voltage dependence of I_p_ is approximately sigmoid, reaching a maximum at positive potentials and approaching zero at very negative potentials. We first measured the maximum turnover rate for the unedited pump and all single edited versions (Figure S2). To estimate this parameter we expressed these constructs in *Xenopus* oocytes and measured I_p_ at positive voltages, where it reaches its maximum, while estimating the pump density in the same oocytes. The maximum turnover rate for the unedited pump is 27.0±4.7 cycles·s^−1^ (at 22°C). None of the rates determined for the edited versions differed significantly from the unedited construct when compared at the same temperature. Thus, at positive voltages, editing has little effect.

**Figure 3:**
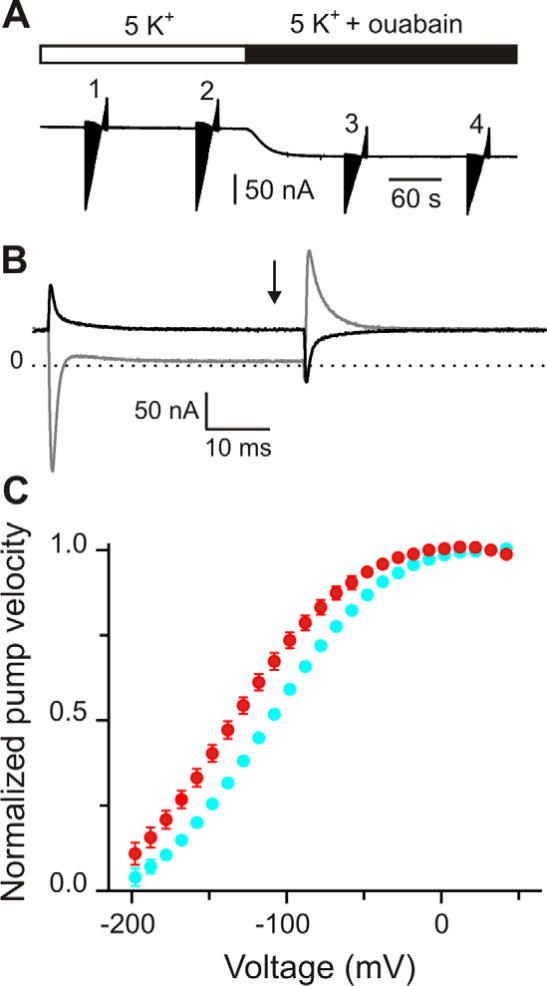
The I877V edit shifts the voltage dependence of the Na^+^/K^+^ pump's turnover rate. Na^+^/K^+^ pump currents were studied using the cut-open oocyte voltage-clamp with *Xenopus* oocytes expressing the unedited and I877V versions of SqNaKα1. (A) Chart recording at a slow sampling rate (1 kHz) of the entire experiment used to measure Na/K pump currents at different voltages. Current-voltage patterns (40 ms steps from −198 mV to 42 mV in 10 mV increments) were recorded twice in a 5 mM K^+^ external solution and then repeated in the same solution with 100 uM ouabain. The stability of the oocyte was monitored by subtracting I-V patterns recorded under the same condition (1–2 and 3–4). (B) Na^+^/K^+^ pump currents were measured by isolating the ouabain sensitive component (2–3) and recording the steady-state value (arrow; average of last 5 ms). The dark trace was recorded at 42 mV and the light trace at −198 mV. Presteady-state transients result from Na^+^-Na^+^ exchange. (C) Current measurements normalized to maximum values for SqNaKα1 (cyan circles; *n* = 6) and SqNa/Kα1 I877V (red circles; *n* = 8). Error bars (s.e.m.) are only shown when they are larger than the symbols.

The Na^+^/K^+^ pump's turnover rate at negative voltages, where it is partially inhibited, is a more relevant measurement because the pump predominantly operates over these potentials. Next we investigated whether editing affects the voltage dependence of the pump's transport velocity. To illustrate our approach, Figure 3A shows a current record of the entire experiment recorded on a slow time scale. The oocyte is held under voltage clamp at 0 mV, where the I_p_ is maximal. The rapid vertical deflections are the current changes in response to 40 ms voltage pulses from the holding potential to various potentials between −198 mV and +42 mV (in 10 mV increments). After each step the voltage was returned to the holding potential. The voltage protocol was repeated in each experimental condition to verify the stability of the preparation. After the application of 100 µM ouabain, the current trace visibly becomes smaller due to inhibition of I_p_. To isolate I_p_, current traces after ouabain application (3) were subtracted from those before (2). Figure 3B shows an example of these traces at the extreme voltages (−198 mV in gray, +42 mV in black). Steady-state I_p_ (arrow) was determined at all voltages by averaging the final 5 ms of each trace, after the transients had settled. Similar measurements were performed for the unedited pump, and all single edited versions. I877V differed substantially from the unedited version. Figure 3C shows the normalized voltage dependence of the pump velocity for I877V and the unedited pump. The principal effect of I877V is to shift the I_p_-V curve ∼25 mV to more negative potentials, thereby relieving voltage dependent inhibition. Because there is ∼2-fold less extracellular Na^+^ in oocyte strength solutions than in those used for squid, both curves would be shifted approximately 60 mV to the right, as we have previously shown [10]. From this we estimate that I877V would significantly increase I_p_ at the resting potential, which is ∼−60 mV in the squid axon [27]. Under physiological conditions the pump's voltage dependence comes mostly from the transitions underlying extracellular Na^+^ release [5],[6],[8],[10],[28]–[30]. Therefore, these results suggest that the I877V edit targets this process.

**Figure 4:**
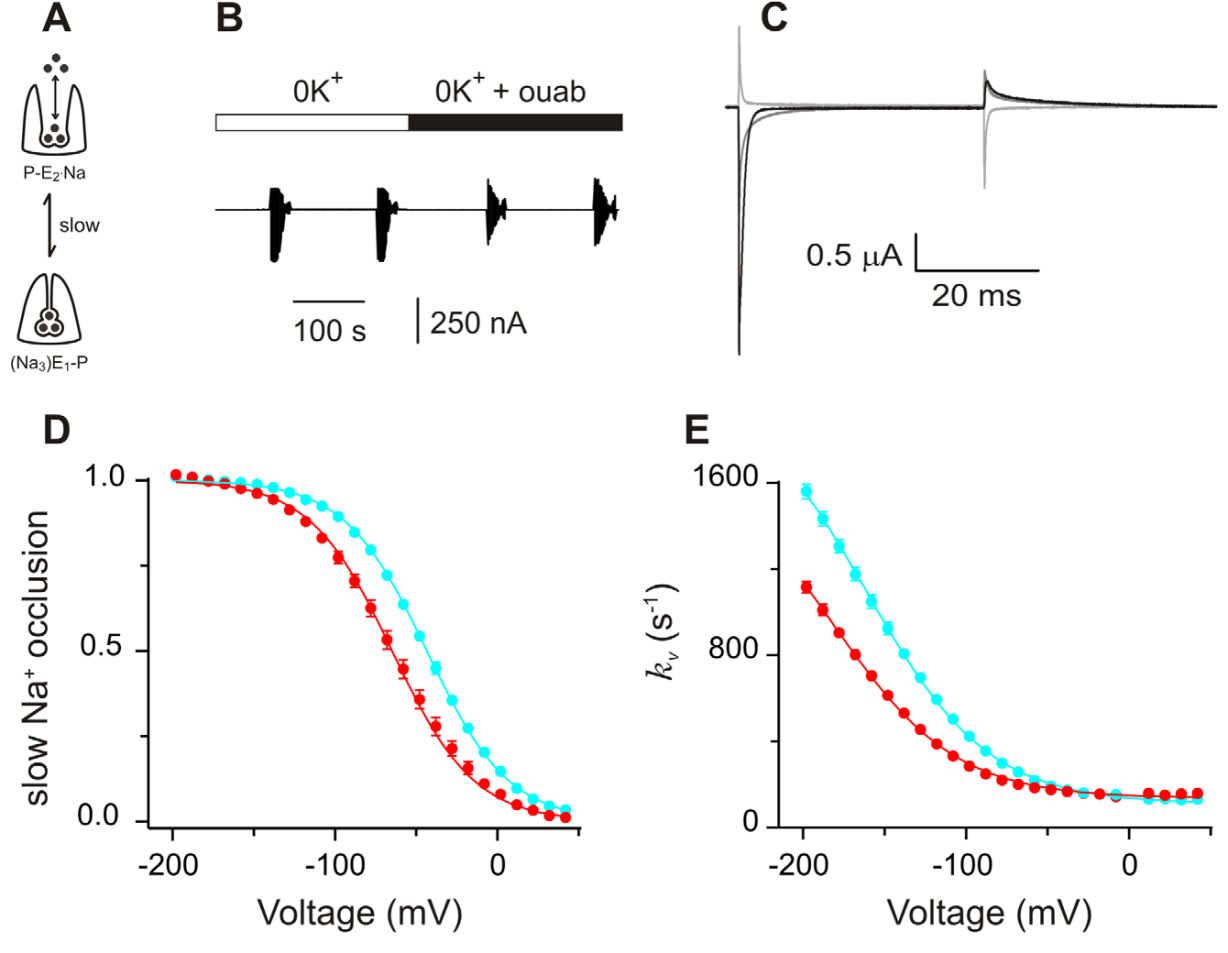
The I877V edit shifts the voltage dependence of Na^+^ release. (A) A simplified pseudo-two-state model for external Na^+^ release. (B) Chart recording at a slow sampling rate (1 kHz) used to illustrate our approach to measuring Na-Na exchange. The pumps are limited to the subset of states shown in part (A) by removing all K^+^ and maintaining a high internal [ATP]. I-V patterns were the same as in the previous figure, and time controls were performed in each condition to assess the stability of the preparation. Transient Na^+^/K^+^ pump currents were isolated by subtracting traces before and after the application of 100 uM ouabain. (C) Transient Na^+^/K^+^ pump currents at −198 mV (black), −78 mV (grey), and 42 mV (light grey). (D) Relative Na^+^ occlusion versus voltage, measured by integrating the slowest component of the off transients from traces like those shown in part (C). The normalized charge moved at each voltage was then fit to a Boltzmann function of the form y = 1/(1+exp(zq(Vq-V)F/RT)), where zq is the apparent valence, Vq is the midpoint, and F, R, and T have their usual meanings and values. Vq, calculated from fits of individual experiments, was −43.5±2.6 mV for SqNa/Kα1 (cyan; ± s.e.m, *n* = 5) and −75.7±3.0 mV for SqNa/Kα1 I877V (red; ± s.e.m, *n* = 5). (E) Voltage dependence of the relaxation rates for Na^+^ occlusion, measured by fitting the slow component of the on transients from traces like those shown in part (C) to a single exponential and then converting the time constant to a rate (*n* = 5 for each construct). These values were then fit to a modified Hill expression of the form:
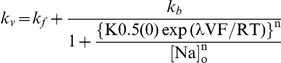
where *k*_f_ and *k*_b_ are voltage-independent forward and backward rate constants, K_0.5_(0) is the apparent affinity for [Na]_o_ at 0 mV, n is the Hill coefficient, λ is the fractional electrical field of the access channel, V is voltage, and F, R, and T have their usual meaning and values. Best fit values for SqNa/Kα1 are: *k*_f_ = 110 s^−1^, *k*_b_ = 1,983 s^−1^, K_0.5_(0) = 7.7 M. For SqNaKα1 I877V they are: *k*_f_ = 136 s^−1^, *k*_b_ = 1,618 s^−1^, K_0.5_(0) = 13.1 M. For each fit the Hill coefficient was constrained to 1 as previously reported [8] and [Na]_o_ was 0.11 M. For SqNaKα1, λ was 0.67. Because the I877V data were shifted negatively and showed no trend towards saturation at negative values, λ was constrained to 0.67 for this construct as well. We were able to validate this assumption independently because the relationship between the voltage-dependence of the Q-V relationship and the concentration of external Na^+^ is proportional to λ. Thus, in a separate set of experiments (not shown) the midpoint of the Q-V relationship for each construct shifted leftward by the same amount by reducing the external [Na] from 110 mM to 55 mM (−35 mV for SqNa/Kα1 and −33 mV for SqNaKα1 I877V), thus demonstrating that λ is the same for both pumps.

**Figure 5:**
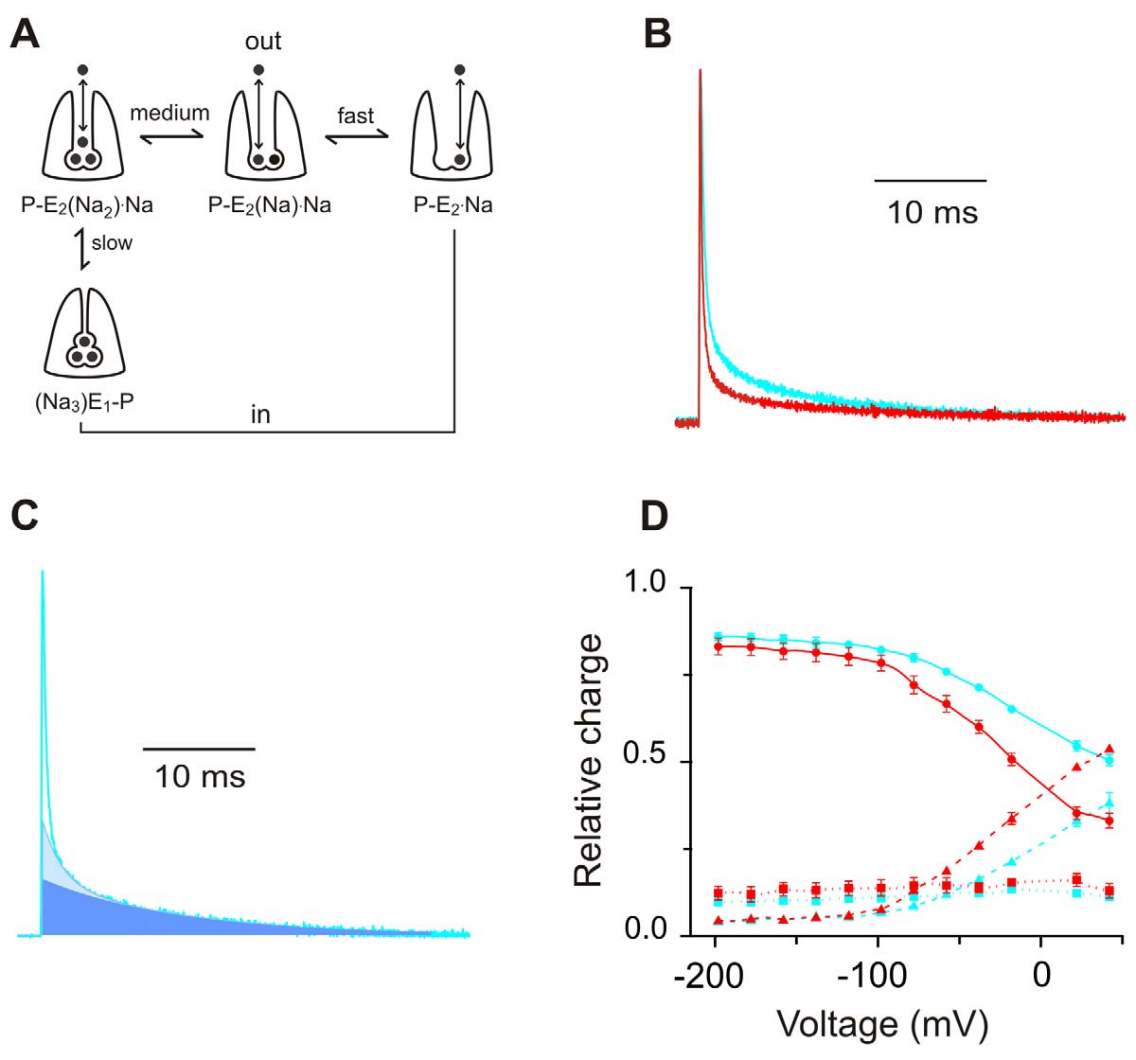
The I877V edit shifts the relative proportion of each charge moving step. (A) A simplified schematic of the Na^+^/K^+^ pump's transport cycle, showing only those states involving extracellular Na^+^ binding/release and occlusion/deocclusion. The fully occluded (Na_3_)E_1_-P state is favored at negative voltages, and the P-E_2_ state, containing no bound Na^+^, is favored at positive voltages. (B) Examples of ouabain subtracted off transients for SqNaKα1 and SqNaKα1 I877V at 0 mV following a prepulse to −58mV. (C) Dissecting distinct components from off transients. Solid blue, shaded blue and transparent areas represent the charge moved by the slow, medium, and fast components, respectively. See Methods for details of how these components were isolated. (D) Proportional charge carried by each component at 0 mV following prepulses to various potentials (slow component = circles and solid line, medium component = squares and dotted line, and fast component = triangles and dashed line). Color scheme is the same as in previous figures.

To better understand the mechanism by which I877V shifts the Na^+^/K^+^ pump's I_p_-V relationship, we studied the process of external Na^+^ binding/release and occlusion/deocclussion in isolation by removing all K^+^ and maintaining the intracellular ATP concentration at high levels (Figure 4A). As before, the membrane was stepped to a wide range of potentials and stability was assessed by repeating voltage protocols in each condition (Figure 4B). Under these ionic conditions ouabain sensitive currents contain only transient components, reflecting the redistribution of external Na^+^ between occluded and deocluded states (see Figure 5A). Examples of these currents for the unedited pump at three potentials are given in Figure 4C. Analysis of these traces shows that there are three kinetic components, as in the squid axon where each is thought to reflect the sequential release of one of the three Na^+^ [6],[8]. We first focused on the slowest component (τ∼12 ms at 0 mV) because it tracks the rate-limiting transition for Na^+^ release and is therefore responsible for determining the I_p_-V relationship's shape. Its voltage-dependence was estimated by integrating the slow component of the off transients and the results are plotted in Figure 4D. As with the steady-state pump currents (Figure 3C), the I877V edit shifts the charge distribution 32 mV towards more negative potentials, indicating that the voltage dependence of the distribution between (Na_3_)E1-P and P-E_2_(Na_2_)Na states has been targeted. Is this due to a change in rates associated with this transition? The rate constants between these two states can be estimated by fitting the kinetics of the slow component to a simple model, derived from a Hill equation, that has been used to describe this transition in pumps from a variety of preparations [5],[8],[10],[31], including the squid clone expressed in *Xenopus* oocytes (Figure 4E). Conceptually, the model reduces Na^+^ release to two basic steps: a slow voltage independent conformational change between the occluded and deoccluded states, and a rapid redistribution of ions across a narrow pore that spans part of the membrane's electrical field, which is the step that renders the process voltage dependent [5],[8],[31]. In this model, the relaxation rates reach asymptotes at extreme voltages. At positive potentials, the relaxations approach the forward rate, while at negative potentials they approach the sum of the forward and backward rates (see legend for Figure 4). The steepness of the curve is largely determined by the electrical depth of the access channel, a value that is unchanged by the I877V edit. Data in Figure 4E show that the forward rates for both constructs reach a similar asymptote at positive voltages and fits to the model indicate that the backwards rates do so as well. The small changes that I877V does cause to these rate constants are not sufficient to account for the shift in the voltage dependence of I_p_. Of greater significance, the model predicts that I877V considerably reduces the apparent affinity for extracellular Na^+^, a change that could be caused by very different physical factors. Because the pump's cation binding sites are thought to be far from position 877, it is unlikely that this edit directly reduces the pump's affinity for Na^+^. In addition, the amino acid change caused by the edit is conservative, making it unlikely that it changes the electrostatics along the ion permeation pathway [32],[33], another mechanism that could plausibly affect the apparent affinity [10],[34]–[37]. An alternative that is more consistent with the amino acid change is that I877V shifts the state occupancy from deeply occluded states towards those that favor release.

Which transitions does I877V affect? Just as the transition from (Na_3_)E1-P↔P-E_2_(Na_2_)·Na can be tracked by the slow component of the relaxations, the transitions between P-E_2_(Na_2_)·Na↔P-E_2_(Na)Na and P-E_2_(Na)·Na↔P-E_2·_Na can be tracked by the medium (τ∼1.5 ms at 0 mV) and fast components (τ<200 µs at 0 mV), respectively (Figure 5A,C). In order to estimate the transition rates between these states we would have to accurately measure the kinetics of each component. In our experimental set-up this is not possible because the time constant of the fast component is comparable to that of the clamp. However, by focusing on the proportion of charge carried by each component in the off transients, we can get a snapshot of the state occupancy during the conditioning pulse (Figure 5C). A visual inspection of off transients following a prepulse to −58 mV shows a clear difference caused by I877V (Figure 5B): the fast component is more pronounced and the slow component is reduced. A more rigorous quantification over a broad range of voltages shows that the reduction of the slow component in I877V comes at the expense of the fast component, a trend that is particularly apparent at positive voltages (Figure 4D). The medium component, on the other hand, carries about the same proportion of charge in both pumps at all voltages. From these data we conclude that I877V selectively targets the release of the last Na^+^ (fast component), thereby shifting the entire equilibrium towards release.

## Discussion

This study demonstrates that RNA editing could have broader physiological impacts than previously supposed, affecting processes outside of fast electrical signaling [14]. Although a large number of editing sites have now been identified in both vertebrates and invertebrates [14],[38], in few cases have their mechanistic consequences been worked out. This is probably because they often create but subtle changes. For example, in a human K^+^ channel editing specifically targets the process of fast inactivation by the removal of a single methyl group in the pore cavity [18]. Here, by making the same change (I→V), editing selectively alters the process of external Na^+^ release, primarily by increasing the occupancy of the states associated with the extracellular release of the final Na^+^. By consequence, the voltage dependence of the transport process is shifted to negative potentials, increasing the Na^+^/K^+^ pump's turnover rate over the physiological range. Taking into account the extent of Na^+^-dependent inhibition for marine osmoconformers (i.e. those that are isotonic with sea water; [10]), from these data we estimate that the I877V edit would cause the Na^+^/K^+^ pump's turnover rate to increase by ∼40% at the resting potential. For an organism, it is particularly important to carefully regulate Na^+^/K^+^ pump function because of the vast quantity of energy that it consumes. Previous reports have hypothesized that RNA editing fine tunes the nervous system by making small, specific changes to protein function [39]–[41] and our results support this idea.

The process of external Na^+^ release is voltage dependent because of the pump's architecture. After unbinding from the protein, Na^+^ must exit through a narrow access channel, not unlike the pore of an ion channel, which spans part of the transmembrane electric field. No matter what the ionic environment, evolution has tuned this structure so that Na^+^/K^+^ pumps are inhibited by extracellular Na^+^ and negative voltages to a similar extent. Why depress activity? An obvious possibility is that the Na^+^/K^+^ pump's voltage dependence allows for the turnover rate to be adjusted. Here, using a heterologous expression system, we show that it is indeed a target. By editing Na^+^/K^+^ pump mRNAs, the turnover rate could be precisely adjusted in different neurons, presumably to meet the specific demands of ion homeostasis. An I877V mutation in the Na^+^/K^+^ pump gene, on the other hand, would uniformly change the physiology of all the pumps it encodes.

The molecular data presented in this study clearly demonstrate that pump structure is being regulated in a tissue specific manner. The idea that these changes alter function in response to metabolic requirements is supported by the I877V edit electrophysiological data from oocytes. This site is robustly edited in multiple regions of the central nervous system, areas composed of small neurons with presumably high rates of firing [42],[43]. I877V is scarcely edited in the giant axon, a structure known to fire at very low rates in vivo [44]. It is worth noting that out of over 250 Na^+^/K^+^ pump sequences from different organisms that we surveyed, all but one have an Isoleucine at this position. Only the sequence from the electric organ of the electric eel, an organ exceptionally rich in Na^+^ channels, has a valine at position 877 [45]. Editing at codons R663 and K666 is also regulated between tissues. Although our electrophysiology approach did not uncover a physiological role for these sites, they could certainly be important for regulating an aspect of Na^+^/K^+^ pump physiology not related to ion transport. In both vertebrates and invertebrates, RNA editing plays an important role in diversifying the protein structure and function. Along with other recent reports, these data show that a surprisingly wide variety of cellular functions can be tuned by editing [46],[47]. Further studies using squid neurons will allow us to directly assess the role that editing plays in regulating ion homeostasis.

## Methods

### Molecular Biology

The initial cloning of the SqNaKα1 cDNA, which was edited at R663G and K666G but not at I877V, has been described in detail in a previous report [10]. In brief, degenerate PCR primers based on conserved regions of Na/K pump α subunits were used to amplify a partial cDNA fragment from *Loligo*. The full-length sequence was determined by 5′ and 3′ RACE and a full-length clone was isolated by PCR using a high fidelity polymerase. For this study, an adult *Loligo opalescens* specimen was collected from Monterey, CA. RNA was extracted from the two giant fiber lobes, which were manually separated from the rest of the stellate ganglion, and used to synthesize cDNA. Full-length SqNaKα1 cDNAs (genbank EF467998) were then amplified using Phusion DNA polymerase (New England Biolabs) and 50 individual clones were sequenced. To isolate the SqNaKα1 gene, genomic DNA was extracted from the gill of the same animal that was used for the cDNA. A genomic library was then made using a Fosmid vector system (EpiFos Copy Control library, Epicentre technologies, UK). To construct the library, ∼40 kb pieces of genomic DNA were size selected by pulsed-field gel electrophoresis, packaged, and then transformed into *E. coli*. Using ^32^P end-labeled oligos complementary to SqNaKα1 cDNA as hybridization probes, a single positive colony was isolated and sequenced to completion. This clone contained all but the first 138 bp of the cDNA sequence and portions of the first intron. The rest of the genomic sequence was isolated by PCR. Mutagenesis was performed by a standard PCR-based strategy using mutant oligonucleotides. All mutants were generated using *Pfu* DNA polymerase and verified by DNA sequencing. The poison primer extension assay used to measure editing efficiencies has been described in detail [26]. The following oligonucleotides, all labeled with 5′ Hexachlorofluorescein, were used for the assay: CATTCCAGTCGATCAAGTTAATTC with AcycloG for R663G, GTCGATCAAGTTAATTCAAGGGA with AcycloA for the first edited adenosine of K666G, TCAAGTCGGTTCCATGGATTACAGCT with AcycloT for the second edited adenosine of K666G, and CGCTGGATTTTTCACCTATTTTG with AcycloG for I877V.

### Electrophysiology Using the Cut-Open Oocyte Vaseline Gap Technique

Functional expression of squid Na^+^/K^+^ pumps in *Xenopus* oocytes has been described before. Na^+^/K^+^ pump charge translocation and current-voltage relationships were studied using the cut-open oocyte technique sampling exclusively from the animal pole. Oocytes were clamped with a Dagan CA-1B high performance oocyte clamp. An Innovative Integrations SBC6711 board with the A4/D4 module and GPATCH software (kindly provided by Dr. Francisco Bezanilla) were used to control voltage and to digitize analog signals. Data were acquired at 100 kHz and filtered at 20 kHz. Intracellular voltage was measured with a 0.2–0.3 MΩ pipette filled with 3M NaCl and bridges were filled with 3M Na-MES in 3% agarose. Oocytes were permeabilized with 0.2% Saponin in internal solution. The internal solution used for all experiments contained (in mM): 80 Na-Glutamate, 20 TEA-Glutamate, 10 MgS0_4_, 10 Hepes, 5 EGTA, 5 Na-ATP, pH 7.5. The 5K external solution contained: 100 Na-Glutamate, 5 K-Glutamate, 5 BaCl_2_, 2 NiCl_2_, 5 HEPES, 2 MgCl_2_, 0.3 Niflumic acid, pH 7.5. The 0 K external solutions contained: 100 Na-Glutamate, 5 NMG-Glutamate, 5 BaCl_2_, 2 NiCl_2_, 5 HEPES, 2 MgCl_2_, 0.3 Niflumic acid, pH 7.5.

### Analysis of Distinct Kinetic Components Associated with Na^+^ Release

Oocytes were stepped from a holding potential of 0 mV to voltages between −198 mV and +42 mV for 40 ms before returning to 0 mV. This protocol was repeated before and after the application of ouabain to yield the ouabain sensitive component. The off transient resulting from the return to 0 mV was used for further analysis. Traces were fit to three exponentials. The charge moved by the slow and medium components was determined by multiplying the time constant and the amplitude obtained from the fits. The fast component was isolated by subtracting the fits of the medium and slow components from the current trace. The charge moved by the fast component was estimated by numerical integration of the subtracted trace. For prepulse voltages between −198 mV and −88 mV, the values of the time constants for the slow and medium components were left as free parameters during the fits. For all other voltages, where the amplitudes of these components are much smaller, the time constants were fixed to their average values between −198 mV and −88 mV.

## Supporting Information

Figure S1 **Editing of SqNaKα1 mRNAs in vitro.** (A) 32 ng full-length SqNaKα1 RNA was incubated with 5 ng recombinant sqADAR2.1A at room temperature for 2 h in Q200 buffer (200 mM K-Glutamate, 50 mM Tris-Glutamate pH 6, 1 mM DTT, 20% glycerol, 0.5 mM PMSF, 0.4 µg/ml Leupeptin, 0.7 µg/ml Pepstatin, 1 U/ul RNAse Block, and 125 ng/ul yeast tRNA). Recombinant sqADAR2.1A was isolated from *Pichia pastoris* as previously described [24]. After the incubation, RNA was converted into cDNA by reverse transcriptase, and the core SqNaKα1, which contains all four editing sites, was amplified by PCR and directly sequenced. The electropherograms of the regions surrounding each editing site are shown in the figure. Blue, cytosine; red, thymidine; green, adenosine; black, guanosine. Arrows indicate the positions of the adenosines that are edited in vivo. All sequences are in the sense orientation except for the R663G/SqADAR2 combination, which is antisense. All sites are edited at low to moderate levels with SqADAR2.1A. Human ADAR2 edits all sites except R663G. (B) A hairpin structure predicted by MFOLD (http://frontend.bioinfo.rpi.edu/applications/mfold/cgi-bin/rna-form1.cgi) that contains the I877V editing site.
(12.37 MB TIF)

Figure S2 **Measurement of the Na^+^/K^+^ pump's maximum turnover rate.** To estimate the turnover rate, both the forward pump current and the number of pumps were measured in the same oocyte. Our approach is outlined using an oocyte expressing SqNaKα1 clamped with the cut-open oocyte technique. The record in (a) shows the entire experiment recorded on a slow time scale. In each external solution, four IV patterns are averaged, and then this regimen is repeated as a time control. The experiment starts with an oocyte being held at 0 mV and with 0 K_out_, limiting pumps from forward pumping. To measure the forward pump current, 5 mM K_out_ is added to fully activate all available pumps (the apparent affinity for K_out_ is ∼1 mM in the presence of 110 mM external Na^+^; unpublished data). The resulting current can be seen as an upward deflection in the steady-state current. The pump IV is measured by subtracting the I-V in 0K_out_ from the I-V in 5K_out_ (2-1); (b) symbols represent steady-state current values, measured at the end of the pulse, after the transient currents have settled. (c) Charge movement during Na^+^/Na^+^ exchange mode. Subtracting the IVs in 0K_out_ before and after the addition of ouabain (3–4) renders presteady-state currents as external Na^+^ are being occluded and deoccluded. Symbols represent the amount of charge moved at each potential, estimated from the integrals of the transient currents. The solid line is a Boltzmann fit, which provides an estimation of the total amount of charge. By comparing the total charge moved with other means of counting pumps, in both guinea pig cardiac myocytes^1^ and *Xenopus* oocytes^2^ it has been shown that each pump moves the equivalent of 1 elementary charge. Thus, maximal turnover rate becomes the forward pump current at potentials more positive than 0 mV divided by the total amount of charge. Using this approach we determined that at 25°C the maximum turnover rate for the unedited pump (SqNaKαG) was 34.0±3.7 s^−1^ (SD, *n* = 3), for the K666G edit it was 33.0±1.1 s^−1^ (SD, *n* = 3), and for the R663G edit it was 31.0±2.1 s^−1^ (SD, *n* = 3). In a separate set of experiments conducted at 22°C we determined that the maximum turnover rate for the unedited pump was 27.0±4.7 s^−1^ (SD, *n* = 4), and for the I877V edit it was 24.2±3.5 s^−1^ (SD, *n* = 4). These data indicate that the maximum turnover rate does not differ significantly between any of these constructs.
(7.85 MB TIF)

## Abbreviations

λ: Fractional electrical distance of the Na^+^/K^+^ pump's access channel
ADAR: Adenosine Deaminase that Acts on RNA
I_p_: Current produced by the Na^+^/K^+^ pump
E1: Na^+^/K^+^ ATPase conformation with ion binding sites facing the intracellular side
E2: Na^+^/K^+^ ATPase conformation with ion binding sites facing the extracellular side
GFL: Giant Fiber Lobe of the Stellate Ganglion (somata of the giant axon)
K_0.5_(0): Apparent affinity for external Na at 0 mV
n: Hill coefficient
(Na): Na occluded
-Na: Na bound but not occluded
OL: Optic Lobe
P-: Phosphorylated
RACE: Rapid Amplification of cDNA Ends
