# Supplementary figures and images for: Regulation of Na+/K+ ATPase Transport Velocity by RNA Editing
Source: PLoS Biol. 2010 Nov 23;8(11):e1000540. doi: 10.1371/journal.pbio.1000540 (PMC2990702; doi:10.1371/journal.pbio.1000540)

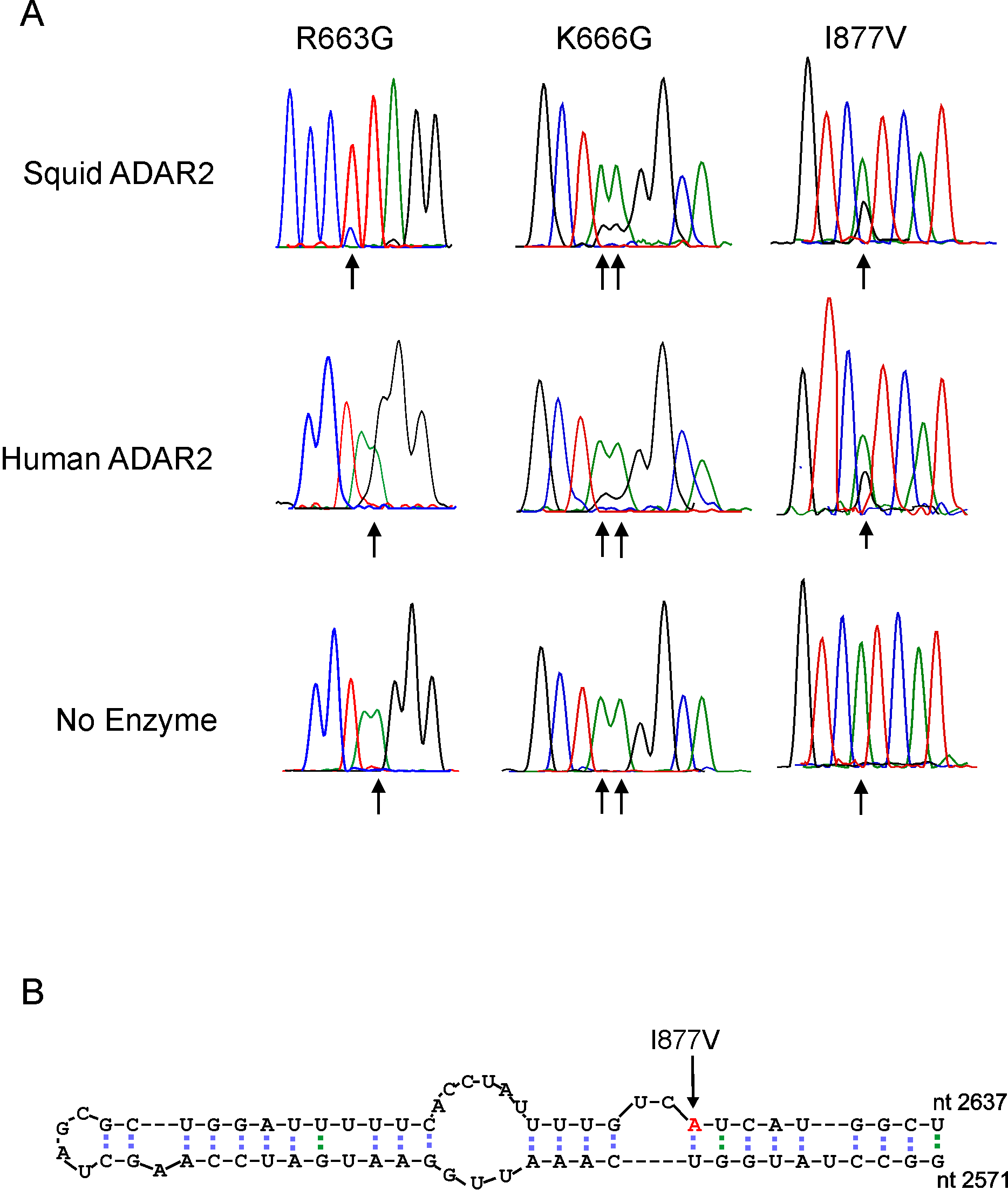

Supplement: Figure S1 — Editing of SqNaKα1 mRNAs in vitro. (A) 32 ng full-length SqNaKα1 RNA was incubated with 5 ng recombinant sqADAR2.1A at room temperature for 2 h in Q200 buffer (200 mM K-Glutamate, 50 mM Tris-Glutamate pH 6, 1 mM DTT, 20% glycerol, 0.5 mM PMSF, 0.4 µg/ml Leupeptin, 0.7 µg/ml Pepstatin, 1 U/ul RNAse Block, and 125 ng/ul yeast tRNA). Recombinant sqADAR2.1A was isolated from Pichia pastoris as previously described [24]. After the incubation, RNA was converted into cDNA by reverse transcriptase, and the core SqNaKα1, which contains all four editing sites, was amplified by PCR and directly sequenced. The electropherograms of the regions surrounding each editing site are shown in the figure. Blue, cytosine; red, thymidine; green, adenosine; black, guanosine. Arrows indicate the positions of the adenosines that are edited in vivo. All sequences are in the sense orientation except for the R663G/SqADAR2 combination, which is antisense. All sites are edited at low to moderate levels with SqADAR2.1A. Human ADAR2 edits all sites except R663G. (B) A hairpin structure predicted by MFOLD (http://frontend.bioinfo.rpi.edu/applications/mfold/cgi-bin/rna-form1.cgi) that contains the I877V editing site. (12.37 MB TIF) [file pbio.1000540.s001.tif]

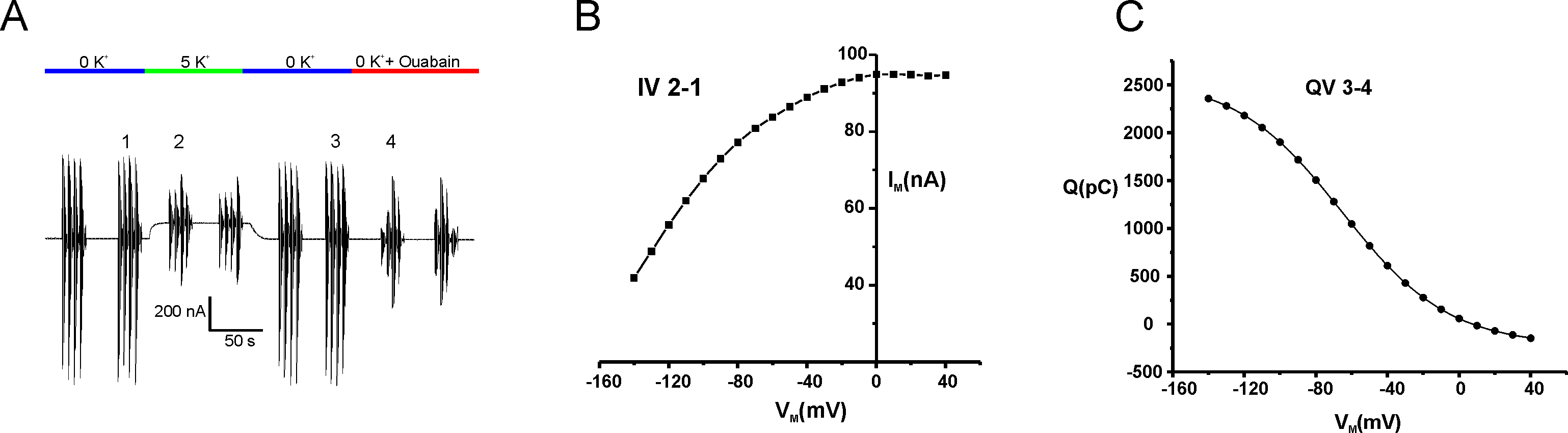

Supplement: Figure S2 — Measurement of the Na+/K+ pump's maximum turnover rate. To estimate the turnover rate, both the forward pump current and the number of pumps were measured in the same oocyte. Our approach is outlined using an oocyte expressing SqNaKα1 clamped with the cut-open oocyte technique. The record in (a) shows the entire experiment recorded on a slow time scale. In each external solution, four IV patterns are averaged, and then this regimen is repeated as a time control. The experiment starts with an oocyte being held at 0 mV and with 0 Kout, limiting pumps from forward pumping. To measure the forward pump current, 5 mM Kout is added to fully activate all available pumps (the apparent affinity for Kout is ∼1 mM in the presence of 110 mM external Na+; unpublished data). The resulting current can be seen as an upward deflection in the steady-state current. The pump IV is measured by subtracting the I-V in 0Kout from the I-V in 5Kout (2-1); (b) symbols represent steady-state current values, measured at the end of the pulse, after the transient currents have settled. (c) Charge movement during Na+/Na+ exchange mode. Subtracting the IVs in 0Kout before and after the addition of ouabain (3–4) renders presteady-state currents as external Na+ are being occluded and deoccluded. Symbols represent the amount of charge moved at each potential, estimated from the integrals of the transient currents. The solid line is a Boltzmann fit, which provides an estimation of the total amount of charge. By comparing the total charge moved with other means of counting pumps, in both guinea pig cardiac myocytes1 and Xenopus oocytes2 it has been shown that each pump moves the equivalent of 1 elementary charge. Thus, maximal turnover rate becomes the forward pump current at potentials more positive than 0 mV divided by the total amount of charge. Using this approach we determined that at 25°C the maximum turnover rate for the unedited pump (SqNaKαG) was 34.0±3.7 s−1 (SD, n = 3), for the K666G edit it w [file pbio.1000540.s002.tif]
